# A Dynamic Model of Drag Force for Catalytic Micromotors Based on Navier–Stokes Equations

**DOI:** 10.3390/mi9090459

**Published:** 2018-09-12

**Authors:** Zhen Wang, Qingjia Chi, Tao Bai, Qiang Wang, Lisheng Liu

**Affiliations:** 1 Hubei Key Laboratory of Theory and Application of Advanced Materials Mechanics, Department of Mechanics and Engineering Structure, Wuhan University of Technology, Wuhan 430070, China; wangzhen@whut.edu.cn (Z.W.); qingjia@whut.edu.cn (Q.C.); whut_baitao@163.com (T.B.); 2 Infrastructure Management Department, Wuhan University of Technology, Wuhan 430070, China; qiang_wang@whut.edu.cn; 3 State Key Laboratory of Advanced Technology for Materials Synthesis and Processing, Wuhan University of Technology, Wuhan 430070, China

**Keywords:** conical micromotor, hydromechanics, Navier-Stokes equation, drag force

## Abstract

In past decades, considerable advances have been achieved in micro and nanomotors. Particular attention has been given to self-propelled catalytic micromotors, which have been widely used in cell separation, drug delivery, microsurgery, lithography and environmental remediation. Fast moving, long life micromotors appear regularly, however it seems there are no solutions yet that thoroughly clarify the hydrodynamic behavior of catalytic micromotors moving in fluid. Dynamic behavior of this kind of micromotors is mainly determined by the driving force and drag force acting on the micromotors. Based on the hydromechanics theory, a hydrodynamic model is established to predict the drag force for a conical micromotor immersed in the flow field. By using the computational fluid dynamics software Fluent 18.0 (ANSYS), the drag force and the drag coefficient of different conical micromotors are calculated. A mathematical model was proposed to describe the relationship among Reynolds numbers *Re*, the ratio *λ*, the semi-cone angle δ and the drag coefficient *C_d_* of the micromotors. This work provides theoretical support and reference for optimizing the design and development of conical micromotors.

## 1. Introduction

Micromotors with good potential in the medical and biological fields have been developed for decades. Efficient and fast micromotors can be applied to environmental chemistry [1,2,3,4], drug delivery [5,6,7], microsurgery [8,9] and cell separation [10,11]. In order to improve the efficiency and velocity of micromotors, various geometries of micromotors with their propulsion mechanisms have been proposed. Bubble-propelled catalytic microjets, which convert chemical energy into kinetic energy, display high speed and efficiency [12,13,14]. Based on Li’s experiments [15], conical micromotors have higher propulsion efficiency than other motors, including Janus microspheres [16,17,18,19], rod micromotors [20,21,22,23], nanowires [24], nanoshell micromotors [25,26] and self-assembly micro/nanomotors [16,27]. A remarkable speed of over 1400 body lengths per second for a tubular nanomotor has been gained by Wang’s group [28]. For bubble-driven tubular micromotors, there are two kinds of forces influencing the movement of micromotors. One is the driving force produced by bubbles and flow field, and the other one is drag force caused by viscosity and pressure of the flow field. The velocity of the micromotor is determined by the balance of the driving force and the drag force [29,30]. According to fluid mechanics theory, fluid resistance of micromotors consists of two parts: Fluid pressure and the flow field viscous resistance. When the Reynolds number is low, the viscous force, caused by the shearing motion of the fluid, plays a major part in drag force [31]. The instantaneous velocity of a microjet changes constantly due to the bubble growing, ejecting and bursting [32]. Nonetheless, for the micromotor motion process, the average velocity can be introduced to evaluate the micromotor speed [33,34]. Therefore, the average velocity of micromotors is used to calculate the drag force. Fluid resistance is dependent upon the physical properties of fluids, geometric parameters of micromotors [35] and the motion of fluids. The original form of drag force on the ellipsoid was first proposed by Cox [36]. The drag force equations including $F_{drag}=\frac{2\pi \mu Lv}{\ln\left(L/R\right)-0.72}$ [29,37] and $F_{drag}=\frac{2\pi \mu Lv}{\ln\left(L/R\right)-0.5}$ [13] are applicable to calculate the drag force on a circular cylinder of finite length and a long spheroid. If the relative motion is along the axis of symmetry, the drag force is given by $F_{drag}=\frac{2\pi \mu Lv}{\ln\left(L/R\right)-0.81}$ [36]. A corrected drag force formula was proposed by Li et al. [15]. Complex shape correction parameters are introduced to describe the drag force of conical micromotors as $F_{drag}=\frac{2\pi \mu Lv}{\ln\left(L/R\right)+C_{1}}$ [38] based on the drag force equations mentioned above. All the modified formulas are used to determine the drag force on the tubular micromotors. There is a problem however, as whilst these formulas confirm the drag force on solid ellipsoid, cylinder or cone frustum, the tubular micromotor designs with a hollow construction adds a complication. The inner surface of the tubular micromotor makes contact with the fluid, meaning that the drag force from the inner surface can’t be neglected.

To study the drag force of conical micromotors, the hydrodynamics theory is applied to the drag force calculation. Navier–Stokes equations and the continuity equation are established for the surrounding flow field [39]. The fluid flows from the front of the micromotor, which simulates the movement of the micromotor at the average velocity. The ANSYS Fluent solver is used to execute computational fluid dynamic (CFD) simulations and calculate the drag force [40]. For the CFD solution, the SIMPLE scheme was used with central spatial differencing [41]. An unstructured mesh was used for all simulations and mesh independence studies were carried out to ensure that the final CFD solution was free of mesh resolution errors [42]. This paper aims to investigate the drag force of conical micromotors, of different geometries, at different average velocities. By using the normalization method, we try to build a dynamic relationship among dimensionless quantities, including the drag coefficient, Reynolds number, semi-cone angle and the rate of length to a larger radius. 

## 2. Theory and Method

**Figure 1:**
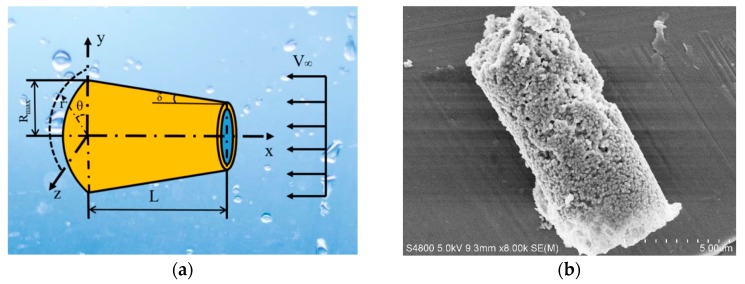
Schematic and SEM image of the micromotor.

When referring to the fluid field, there is an important dimensionless parameter, the Reynolds number (*Re*), which can be used to predict flow patterns in different fluid flow situations. The Reynolds number is the ratio of inertial forces to viscous forces. It can be defined as $R_{e}=\rho vD/\mu$. Here, *ρ* is the density of the fluid, *v* indicates the average velocity of a micromotor, *D* = 2*R*_max_ is the larger diameter of the micromotor as shown in Figure 1a and *μ* is the dynamic viscosity of the fluid.

In this paper, the micromotor moves at a very low Reynolds number since the size of the micromotor is so small. Thus, the viscous resistance is remarkable, which causes a drag force being applied to the micromotor as it moves in the fluid. At a low Reynolds number, the drag force of the micromotor is caused by fluid pressure and viscous resistance. 

Based on the movement of conical micromotors, a rectangular coordinate system is established. The *X*-axis is along the length of the micromotor. The parameters *L*, *δ*, *R*_max_ and *V*_∞_ denote the length, the semi-cone angle, the larger radius of the micromotor and the fluid velocity distance from the micromotor. A cylindrical coordinate system (*r*, *θ*, *x*) is built according to the existing Cartesian coordinate system.

As shown in Figure 1, the azimuthal velocity is negligible in cylindrical coordinates, *V_θ_* = 0. According to the Navier-Stokes and general continuum equations, the relationship between the pressure and velocity of fluid around the micromotor can be described as:

(1) $$\{\begin{array}{c} \frac{V_{r}}{r}+\frac{\partial V_{r}}{\partial r}+\frac{\partial V_{x}}{\partial x}=0\\ \frac{\partial P}{\partial r}=\mu \left(\frac{1}{r}\frac{\partial }{\partial r}\left(r\frac{\partial V_{r}}{\partial r}\right)+\frac{\partial^{2}V_{r}}{\partial x^{2}}-\frac{V_{r}}{r^{2}}\right)\\ \frac{\partial P}{\partial x}=\mu \left(\frac{1}{r}\frac{\partial }{\partial r}\left(r\frac{\partial V_{x}}{\partial r}\right)+\frac{\partial^{2}V_{x}}{\partial x^{2}}\right) \end{array}$$

where *V_r_* is the speed of the flow field in the *r* direction, *V_x_* is the speed of the flow field in the *x* direction, *μ* is the dynamic viscosity of fluid and *P* is the pressure of the fluid. As the Reynolds number is relatively low, the inertial force and gravity of fluid can be neglected. 

The surface of a micromotor is assumed to be a no-slip boundary, and therefore the velocity of fluid satisfies the boundary conditions:

(2) $$V_{r}=0\;V_{x}=0$$

Meanwhile, the fluid velocity at infinity can be written as:

(3) $$V_{r}=0\;V_{x}=V_{\infty }$$

According to the constitutive equation of fluid, the pressure distribution can be gained from velocity distribution of fluid:

(4) $$\{\begin{array}{c} P_{rr}=-P+2\mu \frac{\partial V_{r}}{\partial r}\\ P_{xx}=-P+2\mu \frac{\partial V_{x}}{\partial x}\\ P_{xr}=\mu \left(\frac{\partial V_{r}}{\partial r}+\frac{\partial V_{x}}{\partial x}\right) \end{array}$$

The drag force *F_drag_* can be obtained by integrating pressure distributions at the surface of the micromotor:

(5) $$F_{drag}=\int_{\Omega }\left(P_{xx}+P_{xr}\right)d\Omega$$

where Ω is the surface of the micromotor. The thickness of the micromotor is ignored since it is much smaller than the characteristic diameter. 

In fluid dynamics, the dimensionless drag coefficient can be defined as:

(6) $$C_{d}=\frac{F_{drag}}{\frac{1}{2}\rho Av^{2}}$$

The reference area A is the frontal area of a micromotor on a plane, perpendicular to the flow direction, which is expressed as:

(7) $$A=\pi \left(R_{max}^{2}-{\left(R_{max}-L\tan\delta \right)}^{2}\right)$$

An important feature of the drag coefficient is that it contains a series of dimensionless parameters, including the Reynolds number *Re*, the ratio of length to a larger radius *λ* and a semi-cone angle *δ*. The Navier–Stokes equation used here has no analytical solution as nonlinear terms exist. So, the numerical method is applied to solve the differential equations. We calculate the drag force of the micromotor in *x* direction using the Fluent numerical calculation software.

**Figure 2:**
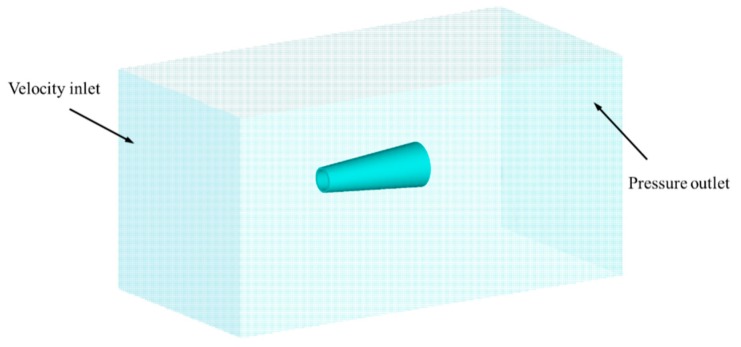
Numerical model and boundaries for simulating the drag force.

The computational domain and boundary conditions used in Fluent are shown in Figure 2. The left side is the velocity inlet boundary, the right side is the pressure outlet boundary, other sides are all wall boundaries. The micromotor was immersed in fluid. The density of the fluid is 998.2 kg/m^3^, the dynamic viscosity is 1.003 mPa·s. The laminar flow model was chosen as the Reynolds number is within a small range.

## 3. Results and Discussions

**Figure 3:**
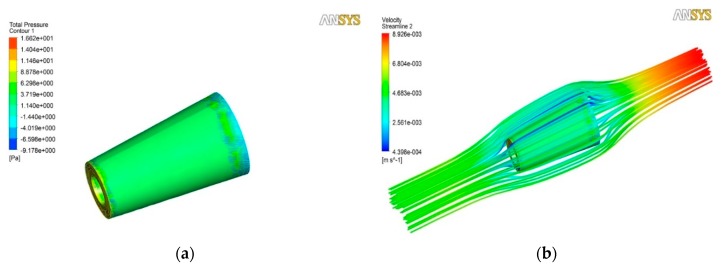
Results calculated by Fluent numerical calculation software. The length of the micromotor is 10 μm. The semi-cone angle is 5°, the larger radius is 5 μm and the inlet velocity of the fluid is 5 mm/s. (**a**) The pressure distribution on the surface of the micromotor; (**b**) the velocity distribution of the flow field around the micromotor.

Computational models corresponding to different semi-cone angles, and the ratios of length to the larger radius for tubular micromotors, have been built and tested at inflow velocities to correspond to different Reynolds numbers. To reach micromotor surface pressure, we assume that the micromotor is fixed and the fluid flows through the micromotor. The drag force on the surface of the micromotor is calculated by integrating the pressure on the micromotor surface in the *x* direction (Figure 3a). According to the control variates method, the simulation models are divided into three groups. 

### 3.1. Calculation Results for Different Numerical Models

**Figure 4:**
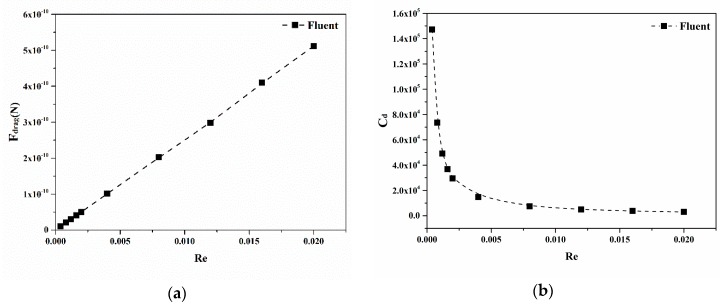
The drag force (**a**) and drag coefficient (**b**) versus the Reynolds number ranging from 4 × 10^−4^ to 2 × 10^−2^.

The calculation results of a drag coefficient for conical micromotors at different Reynolds numbers ranging from 4 × 10^−4^ to 2 × 10^−2^ are presented below. As shown in Figure 4a, the drag force of a conical micromotor increases with the increase of the Reynolds number. In this case, the Reynolds number is especially low (*Re* ≤ 1), so laminar flow occurs, and is characterized by smooth, constant fluid motion. The definition of the Reynolds number generally includes the fluid properties of density and viscosity, plus a velocity and characteristic length. When the fluid is chosen, the density and viscosity of fluid is fixed. So, the Reynolds number is proportional to velocity. As shown in Figure 4a, the increase of the Reynolds number is equal to the increase of velocity, so the drag force on micromotor is proportional to the velocity, with no change to the geometry of the micromotor. This relationship can be justified by the Stokes Law [43]. When the Reynolds number is low, the drag force is approximately proportional to velocity and can be expressed in the form $F_{drag}\propto v$. For a larger Reynolds number, the drag force is approximately proportional to the square of the velocity, $F_{drag}\propto v^{2}$. On the contrary, as pointed out in Figure 4b, the drag coefficient decreases as the Reynolds number increases. From Equation (6), it can be inferred that the drag coefficient is inversely proportional to the velocity. Thus, the drag coefficient decreases when the Reynolds number increases as shown in Figure 4b. The results highlight the different dependencies on the Reynolds number between the drag force and the drag coefficient.

**Figure 5:**
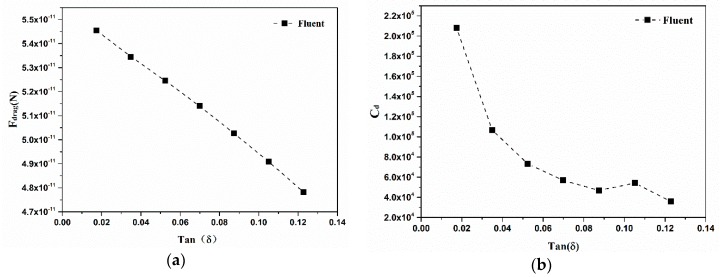
The drag force and drag coefficient versus semi-cone angle ranging from 1° to 7°.

Considering different semi-cone angles of conical micromotors ranging from 1° to 7°, different models are calculated. As depicted in Figure 5a, the drag forces of conical micromotors decrease with the increase of semi-cone angles. The surface area of a micromotor decreases when the semi-cone angle increases, and the drag force decreases due to the reduced integral area. The drag coefficient for conical micromotors also decreases with the increase of a semi-cone angle. The same conclusion has been given in light of Li’s experimental results [15]. The friction force represented by the drag coefficient is proportional to the contact area between the micromotor and the fluid. The surface area decreases with an increase of the semi-cone angle, wherein the larger radius of a micromotor is assumed to be constant. However, as shown in Figure 5b, the slope indicating the relationship between the drag coefficient and the semi-cone angle becomes smaller and smaller, indicating that the semi-cone angle has a greater impact on the drag coefficient when it is small.

**Figure 6:**
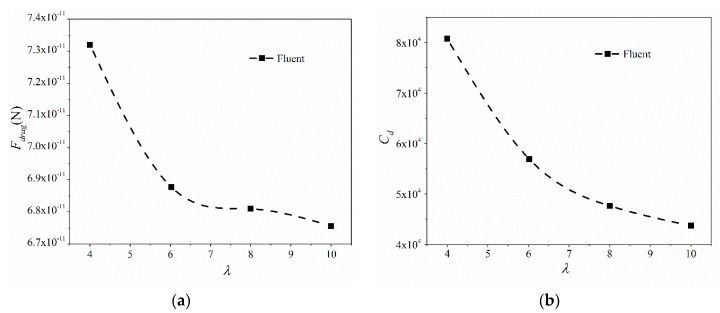
The drag force and drag coefficient versus the rate of length to larger radius ranging from 4 to 7.

Similarly, Figure 6 presents results for the different ratios of length to larger radius, ranging from 4 to 10. Both the drag force and drag coefficient for conical micromotors decrease with the ratio increase. In addition, the decreasing trends become smaller for both drag force and drag coefficient. When the ratio increases from 4 to 10, the larger radius decreases. The velocity increases (40–100 μm/s) in order to make the Reynolds number fixed. At the same time, the surface area decreases as the larger radius decreases. The drag force decreases with the decrease of the larger radius and the velocity increase. From the conclusion gained in Figure 4a, the drag force on a micromotor increases when the velocity increases (100–5000 μm/s) with no change to the micromotor geometry. By contrast, the drag force is more sensitive to geometry than velocity when in a low velocity range. Thus, more attention should be paid to the geometry design in order to get more efficient micromotors in this velocity range.

According to the results, the drag coefficient of a micromotor decreases nonlinearly, along with the increase of the Reynolds number, semi-cone angle and the rate of length to larger radius. These figures demonstrate how geometry and flow field influence the drag force acting on the micromotors. Obviously, the drag coefficient and geometric parameters are nonlinear relationships. And the parameters such as *λ*, *δ* and *Re* are coupled with each other. Through a data-fitting method and analysis, a certain relationship among dimensionless quantities can be obtained. 

### 3.2. Numerical Relationship

The drag force is closely related to the geometry of tubular micromotors [13]. In order to explore a satisfactory understanding of the relationship between dimensionless quantities, it is necessary to analyze the data obtained using Fluent software. The numerical fitting method is applied to the calculation data. 

Influence factors, including the Reynolds number, semi-cone angle and the rate of length to the larger radius, were discussed in the last section. The relationship between dimensionless quantities and the drag coefficients *Re*, tan*δ* and *λ*, have been proposed. Results in Figure 4a show that the drag force is linearly related to the Reynolds number. Data show that the drag coefficient is inversely related to the Reynolds number. In addition, the drag force increases linearly along with the increase of the semi-cone angle. Therefore, the relationship between dimensionless quantities is as:

(8) $$C_{d}=\frac{K\left(\lambda \right)\tan\delta +b\left(\lambda \right)}{Re\left[1-{\left(1-\lambda \tan\delta \right)}^{2}\right]}$$

where *K*(*λ*) and *b*(*λ*) are the functions of the ratio of length to a larger radius *λ*. 

Based on the decreasing trend of drag force and drag coefficient with the ratio *λ* in Figure 6, the expressions of *K*(*λ*) and *b*(*λ*) are assumed as:

(9) $$\{\begin{array}{c} K\left(\lambda \right)=\alpha e^{\beta \lambda }+\frac{\gamma }{\lambda }+\frac{\xi }{\lambda^{2}}\\ b\left(\lambda \right)=\frac{\zeta }{\lambda } \end{array}$$

By combining Equations (8) and (9), the drag coefficient is obtained:

(10) $$C_{d}=\frac{\left(\alpha e^{\beta \lambda }+\frac{\gamma }{\lambda }+\frac{\xi }{\lambda^{2}}\right)\tan\delta +\frac{\zeta }{\lambda }}{Re\left[1-{\left(1-\lambda \tan\delta \right)}^{2}\right]}$$

where *α*, *β*, *γ*, *ξ* and *ζ* are parameters fitted by the nonlinear relationship between the drag coefficient and the ratio. The fitting data are listed in Table 1. 

**Figure 7:**
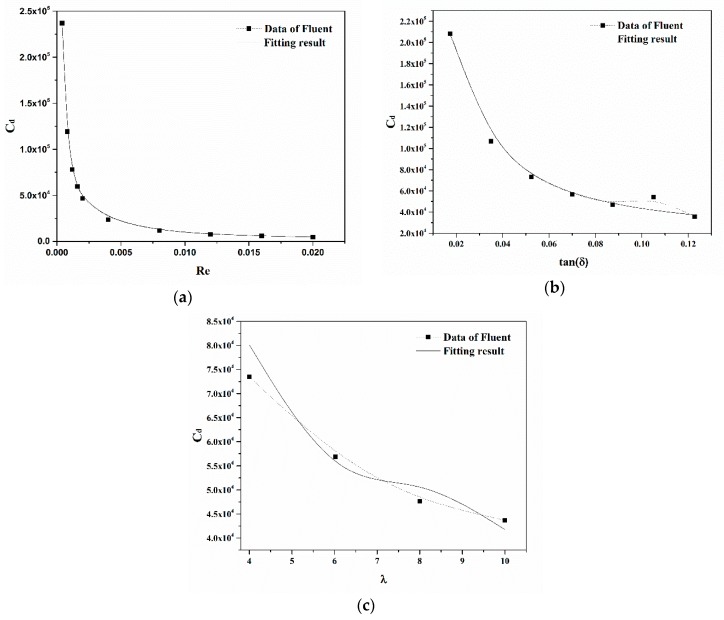
The numerical fitting results of the drag coefficient *C_d_* versus (**a**) *Re*, (**b**) *δ* and (**c**) *λ*.

Correspondingly, Figure 7 gives the numerical fitting results of dimensionless quantities based on Equation (10). These results agree with data gained using Fluent.

To estimate the fitting parameter accuracy, the dimensionless root mean square error is calculated as seen in Equation (11)

(11) $$\varepsilon_{ij}=\sqrt{\frac{1}{N_{j}}\overset{N_{j}}{\underset{i=1}{\sum }}{\left(1-\frac{X_{ij}^{D}}{X_{ij}^{F}}\right)}^{2}}$$

where $\varepsilon_{ij}$ denotes the individual fitting error, *i* denotes the abscissa data point in each curve, *j* refers to the different influence factors: *j* = 1,2,3 corresponds with the Reynolds number *Re*, semi-cone angle tan*δ* and the ratio of length to a larger radius *λ*, respectively. *N_j_* is the number of data points for each curve. $X_{ij}^{D}$ represents the simulation results at the *i-*th abscissa point and for the *j-*th influence factor using Fluent software. In addition, $X_{ij}^{F}$ indicates the fitting result corresponding to the *i-*th abscissa point and for the *j-*th influence factor from the fitted curve. According to the data calculated using Fluent, the following values for *N_j_* can be easily given as: *N*_1_ = 10, *N*_2_ = 7, *N*_3_ = 4.

Furthermore, the global fitting error follows:

(12) $$\varepsilon_{G}=\sqrt{\frac{1}{3}\overset{3}{\underset{j=1}{\sum }}\left[\frac{1}{N_{j}}\overset{N_{j}}{\underset{i=1}{\sum }}{\left(1-\frac{X_{ij}^{D}}{X_{ij}^{F}}\right)}^{2}\right]}$$

The individual fitting errors are listed in Table 2. In addition, the global fitting error is 0.0745.

According to the numerical fitting results, the drag coefficient is inversely related to the Reynolds number, as shown in Figure 7a. Figure 7b gives the relationship between the drag coefficient *C_d_* and *δ* with the same Reynolds number and ratio. Although the drag force increases as the semi-cone angle *δ* increases, the drag coefficient nonlinearly decreases. The reason is that the characteristic area of the micromotor increases with the increase of semi-cone angle. Figure 7c illustrates the effect of the ratio *λ* on the drag coefficient *Cd* for conical micromotors. The drag coefficient nonlinearly decreases as the ratio *λ* increases. Thus, the drag force can be calculated by the formula below:

(13) $$F_{drag}=\frac{\pi }{4}\mu vR_{max}\left[\left(\alpha e^{\beta \lambda }+\frac{\gamma }{\lambda }+\frac{\xi }{\lambda^{2}}\right)\tan\delta +\frac{\zeta }{\lambda }\right]$$

Although simplifications are used, the CFD simulations and the fitting of the data add a small degree of possible variability to the results [44,45]. This model can still be used to predict the drag force of conical micromotors immersed in the fluid field. It also shows that the drag force is only influenced by the micromotor geometry, and the velocity and viscosity of fluid. 

## 4. Conclusions

Based on a simplified motion of conical micromotors, a representative mathematical model was constructed. A numerical method was introduced to solve inhomogeneous partial differential equations. Results calculated using numerical software, show that the drag force increases linearly with the increase of the Reynolds number. However, the drag coefficient decreases nonlinearly as the Reynolds number increases. Meanwhile, the drag force decreases linearly with the increase of the semi-cone angle *δ*, while the drag coefficient decreases nonlinearly. Furthermore, both the drag force and the drag coefficient decrease nonlinearly with the increase of the ratio *λ*. 

In summary, a numerical model used to describe the relationship between dimensionless quantities, including *C_d_*, *Re*, *δ* and *λ*, has been built. According to the results above, they agree with the numerical results produced using Fluent. The aim of our work was to reduce the drag force and increase the velocity of conical micromotors by optimizing the geometry of motors. Based on the numerical results, the drive efficiency can be improved by increasing the semi-cone angle *δ* and the ratio *λ* at the same Reynolds number. The drag force increases with the increase of the Reynolds number. That is to say, as the speed of conical micromotors rises, the drag force also increases. Greater driving force is required to overcome the drag force at the high-speed movement of conical micromotors. Samples have been made, and further experiments on tubular micromotor drag force will be conducted in the future.

## Figures and Tables

**Table 1:** Fitting parameters of the model.

Model Parameters	Values
*α*	34,543.88
*β*	−0.08
*γ*	−175,790.03
*ξ*	307,718.72
*ζ*	350.84

**Table 2:** Fitting errors for different influence factors.

Influence Factor	Individual Fitting Error
*R_e_*	0.0165
tan*δ*	0.0901
*λ*	0.0909

## References

[1] Ma X., Hahn K., Sanchez S. (2015). Catalytic mesoporous janus nanomotors for active cargo delivery. J. Am. Chem. Soc..

[2] Balasubramanian S., Kagan D., Jack Hu C., Campuzano S., Lobo-Castañon M.J., Lim N., Kang D.Y., Zimmerman M., Zhang L., Wang J. (2011). Micromachine-enabled capture and isolation of cancer cells in complex media. Angew. Chem. Int. Ed..

[3] Li T., Li L., Song W., Wang L., Shao G., Zhang G. (2015). Self-propelled multilayered microrockets for pollutants purification. ECS J. Solid State Sci. Technol..

[4] Soler L., Magdanz V., Fomin V.M., Sanchez S., Schmidt O.G. (2013). Self-propelled micromotors for cleaning polluted water. ACS Nano.

[5] Gao W., Kagan D., Pak O.S., Clawson C., Campuzano S., Chuluun-Erdene E., Shipton E., Fullerton E.E., Zhang L., Lauga E. (2012). Cargo-towing fuel-free magnetic nanoswimmers for targeted drug delivery. Small.

[6] Gao W., Wang J. (2014). Synthetic micro/nanomotors in drug delivery. Nanoscale.

[7] Xing M., Yun Z., Kee Woei N., Yanli Z. (2013). Integrated hollow mesoporous silica nanoparticles for target drug/siRNA co-delivery. Chem. A Eur. J..

[8] Xi W., Solovev A.A., Ananth A.N., Gracias D.H., Sanchez S., Schmidt O.G. (2013). Rolled-up magnetic microdrillers: Towards remotely controlled minimally invasive surgery. Nanoscale.

[9] Flynn A.M., Udayakumar K.R., Barrett D.S. Tomorrow’s Surgery: Micromotors and Microrobots. https://dspace.mit.edu/bitstream/handle/1721.1/41509/AI_WP_337.pdf?sequence=4.

[10] Kagan D., Campuzano S., Balasubramanian S., Kuralay F., Flechsig G.U., Wang J. (2011). Functionalized micromachines for selective and rapid isolation of nucleic acid targets from complex samples. Nano Lett..

[11] Xu T., Soto F., Gao W., Dong R., Garcia-Gradilla V., Magaña E., Zhang X., Wang J. (2015). Reversible swarming and separation of self-propelled chemically powered nanomotors under acoustic fields. J. Am. Chem. Soc..

[12] Wang L., Li T., Li L., Wang J., Song W., Zhang G. (2015). Microrocket based viscometer. ECS J. Solid State Sci. Technol..

[13] Wei G., Sirilak S., Jahir O., Joseph W. (2011). Highly efficient catalytic microengines: Template electrosynthesis of polyaniline/platinum microtubes. J. Am. Chem. Soc..

[14] Gao W., Sattayasamitsathit S., Wang J. (2012). Catalytically propelled micro-/nanomotors: How fast can they move?. Chem. Rec..

[15] Li L.Q., Wang J.Y., Li T.L., Song W.P., Zhang G.Y. (2015). A unified model of drag force for bubble-propelled catalytic micro/nano-motors with different geometries in low Reynolds number flows. J. Appl. Phys..

[16] Wei G., Pei A., Feng X., Hennessy C., Wang J. (2013). Organized self-assembly of Janus micromotors with hydrophobic hemispheres. J. Am. Chem. Soc..

[17] Araki T., Fukai S. (2015). Controlled motion of Janus particles in periodically phase-separating binary fluids. Soft Matter.

[18] Zhang Q., Dong R., Chang X., Ren B., Tong Z. (2015). Spiropyran-decorated SiO_2_-Pt Janus micromotor: Preparation and light-induced dynamic self-assembly and disassembly. ACS Appl. Mater. Interfaces.

[19] Zhang J., Zheng X., Cui H., Silber-Li Z. (2017). The self-propulsion of the spherical Pt-SiO_2_ janus micro-motor. Micromachines.

[20] Wei W., Li S., Lamar M., Suzanne A., Huang T.J., Mallouk T.E. (2014). Acoustic propulsion of nanorod motors inside living cells. Angew. Chem..

[21] Zacharia N.S., Sadeq Z.S., Ozin G.A. (2009). Enhanced speed of bimetallic nanorod motors by surface roughening. Chem. Commun..

[22] Paxton W.F., Kistler K.C., Olmeda C.C., Sen A., Angelo S.K.S., Cao Y., Mallouk T.E., Lammert P.E., Crespi V.H. (2004). Catalytic nanomotors: Autonomous movement of striped nanorods. J. Am. Chem. Soc..

[23] Kovtyukhova N.I. (2008). Toward understanding of the propulsion mechanism of rod-shaped nanoparticles that catalyze gas-generating reactions. J. Phys. Chem. C.

[24] Fournier-Bidoz S., Arsenault A.C., Manners I., Ozin G.A. (2005). Synthetic self-propelled nanorotors. Chem. Commun..

[25] Huang W., Manjare M., Zhao Y. (2013). Catalytic nanoshell micromotors. J. Phys. Chem. C.

[26] Zhao G., Pumera M. (2014). Geometric asymmetry driven Janus micromotors. Nanoscale.

[27] Ning H., Zhang Y., Zhu H., Ingham A., Huang G., Mei Y., Solovev A.A. (2018). Geometry design, principles and assembly of micromotors. Micromachines.

[28] Gao W., Sirilak S., Aysegul U., Allen P., Adam P., Joseph W. (2012). Polymer-based tubular microbots: Role of composition and preparation. Nanoscale.

[29] Fomin V.M., Hippler M., Magdanz V., Soler L., Sanchez S., Schmidt O.G. (2014). Propulsion mechanism of catalytic microjet engines. IEEE Trans. Robot..

[30] Mei Y., Solovev A.A., Samuel S., Schmidt O.G. (2011). Rolled-up nanotech on polymers: From basic perception to self-propelled catalytic microengines. Chem. Soc. Rev..

[31] Wang Z., Chi Q., Liu L., Liu Q., Bai T., Wang Q. (2017). A viscosity-based model for bubble-propelled catalytic micromotors. Micromachines.

[32] Manjare M., Yang B., Zhao Y.P. (2012). Bubble driven quasioscillatory translational motion of catalytic micromotors. Phys. Rev. Lett..

[33] Manjare M., Yang B., Zhao Y.P. (2013). Bubble-propelled microjets: Model and experiment. J. Phys. Chem. C.

[34] Wang H., Moo J.G., Pumera M. (2014). Tissue cell assisted fabrication of tubular catalytic platinum microengines. Nanoscale.

[35] Hong W., Moo J.G.S., Pumera M. (2016). From nanomotors to micromotors: The Influence of the size of an autonomous bubble-propelled device upon its motion. ACS Nano.

[36] Cox R.G. (1970). The motion of long slender bodies in a viscous fluid part 1. General theory. J. Fluid Mech..

[37] Li J.X., Huang G.S., Ye M.M., Li M.L., Liu R., Mei Y.F. (2011). Dynamics of catalytic tubular microjet engines: Dependence on geometry and chemical environment. Nanoscale.

[38] Li L., Wang J., Li T., Song W., Zhang G. (2014). Hydrodynamics and propulsion mechanism of self-propelled catalytic micromotors: Model and experiment. Soft Matter.

[39] Sarkis B., Folio D., Ferreira A.E.F. Catalytic tubular microjet propulsion model for endovascular navigation. Proceedings of the IEEE International Conference on Robotics and Automation.

[40] ANSYS FLUENT 12.0 Theory Guide 2009. http://www.afs.enea.it/project/neptunius/docs/fluent/html/th/main_pre.htm.

[41] Launder B.E., Spalding D.B. (1983). PAPER 8—The numerical computation of turbulent flows. Numerical Prediction of Flow Heat Transfer Turbulence and Combustion.

[42] Ferziger J.H., Perić M. (1999). Computational Methods for Fluid Dynamics.

[43] Clancy L.J. (1975). Aerodynamics.

[44] Mishra A.A., Iaccarino G., Duraisamy K. (2016). Sensitivity of flow evolution on turbulence structure. Phys. Rev. Fluids.

[45] Iaccarino G., Mishra A.A., Ghili S. (2017). Eigenspace perturbations for uncertainty estimation of single-point turbulence closures. Phys. Rev. Fluids.

